# Sedation at the end of life - a nation-wide study in palliative care units in Austria

**DOI:** 10.1186/s12904-016-0121-8

**Published:** 2016-05-14

**Authors:** Sophie Schur, Dietmar Weixler, Christoph Gabl, Gudrun Kreye, Rudolf Likar, Eva Katharina Masel, Michael Mayrhofer, Franz Reiner, Barbara Schmidmayr, Kathrin Kirchheiner, Herbert Hans Watzke, on behalf of the AUPACS (Austrian Palliative Care Study) Group

**Affiliations:** Clinical Division of Palliative Care, Department for Internal Medicine I, Medical University of Vienna, Waehringer Guertel 18-20, 1090 Vienna, Austria; Palliative Support Team, Landesklinikum Horn, Austria; Mobile Hospice and Palliative Care Team, Tiroler Hospizgemeinschaft, Innsbruck, Austria; Department of Internal Medicine, University Hospital Krems, Krems, Austria; Department of Anaesthesiology and Intensive Medicine, Interdisciplinary Center of Pain Therapy and Palliative Medicine, General Hospital Klagenfurt, Klagenfurt, Austria; Department for Palliative Care, Salzkammergut-Klinikum, Vöcklabruck, Austria; Department of Internal Medicine, Krankenhaus der Elisabethinen, Graz, Austria; Department of Radiation Oncology, Comprehensive Cancer Center, Medical University of Vienna, Vienna, Austria

**Keywords:** Palliative care, Sedation, Symptom management

## Abstract

**Background:**

Sedation is used to an increasing extent in end-of-life care. Definitions and indications in this field are based on expert opinions and case series. Little is known about this practice at palliative care units in Austria.

**Methods:**

Patients who died in Austrian palliative care units between June 2012 and June 2013 were identified. A predefined set of baseline characteristics and information on sedation during the last two weeks before death were obtained by reviewing the patients’ charts.

**Results:**

The data of 2414 patients from 23 palliative care units were available for analysis. Five hundred two (21 %) patients received sedation in the last two weeks preceding their death, 356 (71 %) received continuous sedation until death, and 119 (24 %) received intermittent sedation. The median duration of sedation was 48 h (IQR 10–72 h); 168 patients (34 %) were sedated for less than 24 h. Indications for sedation were delirium (51 %), existential distress (32 %), dyspnea (30 %), and pain (20 %). Midazolam was the most frequently used drug (79 %), followed by lorazepam (13 %), and haloperidol (10 %). Sedated patients were significantly younger (median age 67 years vs. 74 years, *p* ≤ 0.001, *r* = 0.22), suffered more often from an oncological disease (92 % vs. 82 %, *p* ≤ 0.001, φ = 0.107), and were hospitalized more frequently (94 % vs. 76 %, *p* ≤ 0.001, φ = 0.175). The median number of days between admission to a palliative care ward/mobile palliative care team and death did not differ significantly in sedated versus non-sedated patients (10 vs. 9 days; *p* = 0.491).

**Conclusion:**

This study provides insights into the practice of end-of-life sedation in Austria. Critical appraisal of these data will serve as a starting point for the development of nation-wide guidelines for palliative sedation in Austria.

## Background

Adequate symptom relief is a central aspect of medical care in all patients, especially those with incurable diseases [1]. However, satisfactory control of symptoms may be difficult as an illness progresses and patients approach the end of life [1–3]. Despite intensive efforts to manage such problems, physical or psycho-existential symptoms may remain uncontrollable in some patients [1, 3, 4]. Therefore, sedation at the end of life is a valid and increasingly used therapeutic intervention to relieve the burden of severe and refractory symptoms [1, 5–7].

According to the European Association for Palliative Care (EAPC), palliative sedation (PS) is defined as the ‘*monitored use of medications intended to induce a state of decreased or absent awareness (unconsciousness) in order to relieve the burden of otherwise intractable suffering..’* [6]. Importantly, PS should be used as last resort at the end of a patient’s life, with the intent of minimizing symptoms in a manner that is ethically acceptable to the patient, caregivers and all healthcare providers involved in the sedation process [6, 7].

Although PS is used increasingly often in terminally ill patients, healthcare professionals are still confronted with numerous clinical and ethical challenges [1, 8]. Besides, literature indicates a considerable heterogeneity with regard to the definition of PS, its frequency, and indications for its use [9, 10].

In recent years, a number of international medical associations [6, 11], national bodies [12, 13], and local institutions [14, 15] have tried to develop guidelines and policies with the aim of defining PS [9, 16]. To date however, no randomized studies have been focused on these aspects and current assertions are based on expert opinions and case series. Whereas the practice of PS has been a subject of research in many countries [17–22], it has not been investigated systematically in Austria.

The present study included the data of more than 2424 patients being cared for at Austrian palliative care wards and mobile palliative care teams. The data were analyzed with regard to the prevalence of intermittent and continuous sedation therapy within the last two weeks of the patients’ lives. In addition we compared sedated and non-sedated patients in terms of clinical characteristics and the duration of admission to a palliative care ward or treatment by a mobile palliative care team.

## Methods

### Setting and participants

The study was performed as a retrospective chart review in palliative care wards and mobile care teams in Austria. Data were obtained from the medical records of patients who had died at one of the palliative care units between 1st June 2012 and 31st May 2013. Each palliative care unit was asked to state the number of patients treated in the study period, the number of patients who had died, and the number of patients with available medical records.

The observation period encompassed the last two weeks of the patients’ lives. Clinical characteristics (age, sex, underlying disease: oncological vs. non-oncological, date of admission and death) as well as co-medication (non-opioids, opioids, antibiotics, deep venous thrombosis prophylaxis, artificial hydration and nutrition) given during the last three days before death were recorded for all patients.

Sedation therapy at the end of life was defined as any sedating intervention initiated in the last two weeks of the patient’s life and given continuously until his/her death (minimal duration one hour), or as intermittent sedation for more than 24 h, even when it was not given at the time of the patient’s death. In order to exclude sedation as a side effect of other medications acting on the central nervous system (such as sleeping medication or anticonvulsants), the palliative care units were instructed upfront at a meeting, informed of the study protocol, and sent an accompanying letter.

Sedation practice in each patient was recorded in a three-page case report form (CRF). Each palliative care unit had to mention whether they had an instruction manual for indications, procedure, and monitoring at their site.

The following parameters were recorded in patients who received sedation: indication(s) for sedation, medication, continuity of sedation (continuous versus intermittent), route of administration (intravenous, subcutaneous), dose (in the first and last 24 h of sedation) and the total duration of sedation (days/hours).

The study protocol was approved by the ethics committee of the Medical University of Vienna (2060/2013).

### Statistical analysis

Categorical data are presented descriptively with absolute numbers and percentages; missing values were below 1 % unless specified otherwise.

For metric data, the median and interquartile ranges (lower and upper quartiles) are reported, based on skewed distribution.

Categorical data were tested using Pearson’s Chi-square tests, metric data with the parameter-free Mann–Whitney *U*-test in independent samples, and Wilcoxon’s matched-pair signed-rank test in dependent samples. The significance level was set two-sided at 5 %; p-values were corrected for multiple tests with the Bonferroni-Holm method. For the estimation of effect sizes in significant results, Pearson’s correlation coefficient (r) or the phi coefficient (φ) are reported, based on the following ratings: 0.1 = small, 0.3 = moderate, and 0.5 = large effect size.

For time to event analysis, the days between admission to a palliative care ward/start of care by a mobile palliative care team and death were compared between sedated and non-sedated patients using the Breslow (generalized Wilcoxon) test [23]. In a subgroup analysis in order to find a potential difference between patients receiving continuous or intermittent sedation the Breslow (generalized Wilcoxon) test was also used.

Analysis was performed with the Statistical Package for the Social Sciences IBM SPSS v.22 (Armonk, NY: IBM Corp).

## Results

**Fig. 1:**
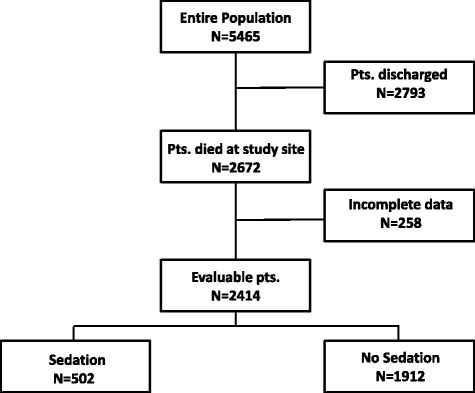
CONSORT diagram of the study population

Twenty-three of 31 (74 %) palliative care wards and mobile care teams in Austria participated in this study. A total of 5465 patients were treated between June 2012 and June 2013 at these units. 2672 (49 %) patients died during their hospital stay or the time period treated by a mobile palliative care team and were therefore eligible for the evaluation of sedation during the last two weeks of their lives. The data of 2414 (90 %) deceased patients were available and could be included in the subsequent analysis (Fig. 1).

### Patient characteristics: overall cohort

The patients’ median age was 73 years (IQR 64–82 years); 52 % were female. One thousand nine hundred thirty (80 %) patients were treated at hospital-based palliative care wards while 484 (20 %) were treated at home by mobile palliative care teams.

The median duration from admission to a palliative care ward/mobile palliative care team until death was 9 days (IQR 4–20 days). Ninety-four patients (4 %) died on the day of admission to a palliative care ward/their first contact with the mobile palliative care team. Two thousand forty two patients (85 %) underwent care at the palliative care ward during the last month before their death or were treated for at least one month by the mobile palliative care team. Two thousand twenty seven patients (84 %) had a malignant disease (Table 1).

**Table 1:** Patients characteristics of the overall cohort and the sedated versus non-sedated patients; missing data range <1

	Overall cohort (*N* = 2414)		Sedated patients (*n* = 502, 21 %)		Non-sedated patients (*n* = 1912, 79 %)		*p*-value	Corrected *p*-value	effect size
Age in years in median (IQR)	73 (64–82)		67 (56–75)		74 (65–83)		≤0.001*	≤0.001*	r = 0.220
Time between admission and death in days in median (IQR)	9 (4 – 20)		10 (5–19)		9 (4–22)		0.491	0.491	-
	n	%	n	%	n	%			
Sex							0.014*	0.070	φ = 0.051
Female	1251	52 %	236	47 %	1015	53 %			
Male	1158	48 %	266	53 %	892	47 %			
Oncological disease	2027	84 %	461	92 %	1566	82 %	≤0.001*	≤0.001*	φ = 0.107
Other diseases	372	15 %	40	8 %	332	17 %			
Type of palliative care							≤0.001*	≤0.001*	φ = 0.175
Hospital based	1930	80 %	470	94 %	1460	76 %			
Mobile	484	20 %	32	6 %	452	24 %			
Co-medication in the last three days of life									
Opioids pain medication	2213	92 %	484	96 %	1729	90 %	≤0.001*	≤0.001*	φ = 0.100
Non-opioid pain medication	1271	53 %	240	48 %	1031	54 %	0.032*	0.128	φ = 0.044
I.v. hydration	1018	42 %	242	48 %	776	41 %	0.001*	0.006*	φ = 0.077
Artificial nutrition	624	26 %	114	23 %	510	27 %	≤0.001*	≤0.001*	φ = 0.099
Antibiotic treatment	404	17 %	96	19 %	308	16 %	0.088	0.176	
DVTP medication	571	24 %	133	27 %	438	23 %	0.071	0.213	

*Abbreviations*: *N* number, *IQR* interquartile range, *I.v.* intravenous, *DVTP* deep venous thrombosis prophylaxis

**Fig. 2:**
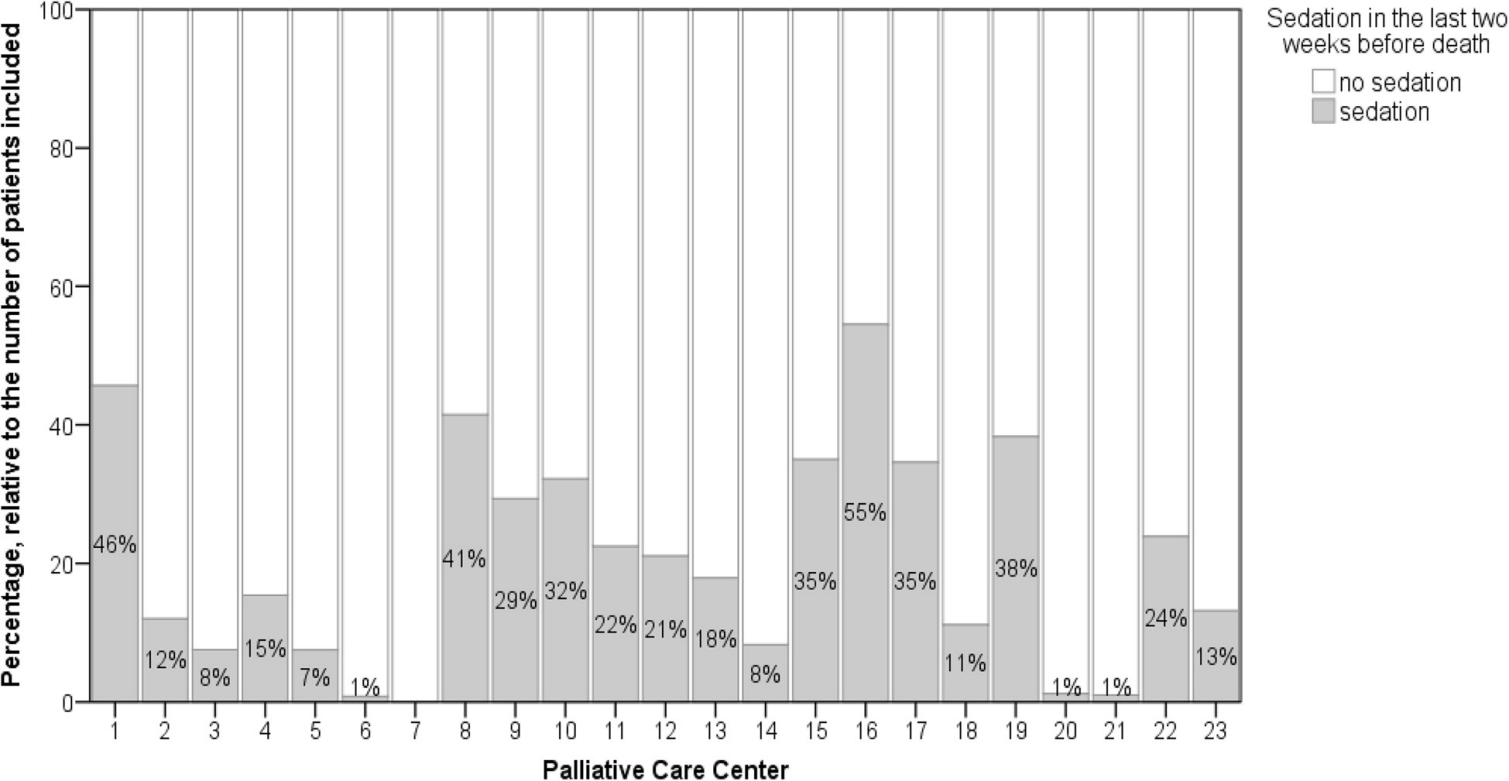
Sedation prevalence in the participating Austrian palliative care centers

In the last two weeks before death, 502 patients (21 %) were sedated. The percentage of sedated patients varied markedly across the participating study centers (range 0–54 %; Fig. 2). Further characteristics in respect of the patients’ co-medication in the last three days of their lives are shown in Table 1.

### Differences between sedated and non-sedated patients

Sedated patients were significantly younger (median age 67 years vs. 74 years, *p* ≤ 0.001, *r* = 0.22), suffered more often from oncological disease (92 % vs. 82 %, *p* ≤ 0.001, φ = 0.107), and were hospitalized more often (94 % vs. 76 %, *p* ≤ 0.001, φ = 0.175). Sedated patients received significantly more opioids (96 % vs. 90 %, *p* ≤ 0.001, φ = 0.100), more intravenous hydration (48 % vs. 41 %, *p* = 0.006, φ = 0.077), and less artificial nutrition (23 % vs. 27 %, *p* ≤ 0.001, φ = 0.099). The details are listed in Table 1.

**Fig. 3:**
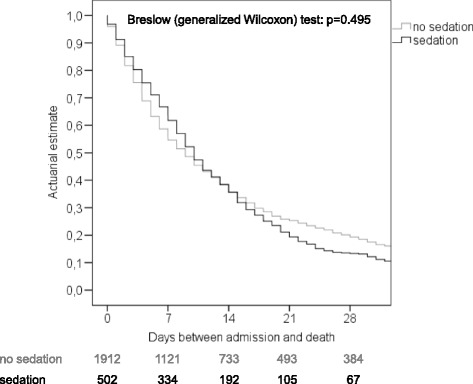
Actuarial Kaplan-Meier estimates in 2414 patients with or without sedation

The median number of days from admission to a palliative care ward/start of care by a mobile palliative care team until death did not differ significantly between sedated and non-sedated patients (10 vs. 9 days; *p* = 0.491; Fig. 3). In a subgroup analysis, no difference was observed between patients receiving continuous and intermittent sedation regarding the number of days from admission to a palliative care ward/start of a mobile care team until death (*p* = 0.259).

### Documentation of sedation at study sites

Twenty-one contributing palliative care units (91 %) used an explicit documentation form for sedation, 14 (67 %) followed a written instruction manual, and 6 (29 %) a written monitoring form.

### Sedation practice in Austria

Continuous sedation until death was given to 356 (71 %) of all sedated patients, and intermittent sedation to 119 (24 %) (missing information for 5 %). The median duration of sedation was 48 h (IQR 10–72 h); 168 patients (34 %) were sedated for less than 24 h and five patients for more than three weeks.

Indications for sedation were delirium (*n* = 254, 51 %), existential distress (*n* = 159, 32 %), dyspnea (*n* = 151, 30 %), pain (*n* = 99, 20 %), and other individual reasons (n = 56, 11 %) (Table 2). Two or three indications for sedation were documented in 36 and 8 % of the patients, respectively.

**Table 2:** Characteristics of sedation in sedated patients (*n* = 502)

			Missing information (%)
Median sedation time in hours (IQR)	48 (10–72)		12 (2 %)
	n	%	13 (3 %)
Type of sedation			27 (5 %)
Continuous	356	71 %	
Intermittent	119	24 %	
Indication for sedation			
Delirium	254	51 %	18 (4 %)
Existential distress	159	32 %	18 (4 %)
Dyspnea	151	30 %	17 (3 %)
Pain	99	20 %	18 (4 %)
Other individual reasons	56	11 %	
Not verifiable	34	7 %	17 (3 %)
Drug used for sedation			
Midazolam	395	79 %	2 (<1 %)
Diazepam	14	3 %	2 (<1 %)
Propofol	13	3 %	2 (<1 %)
Propthipendyl	3	1 %	2 (<1 %)
Levomepromazin	17	3 %	2 (<1 %)
Dihydrobenzperidol	2	<1 %	2 (<1 %)
Lorazepam	66	13 %	2 (<1 %)
Clonazepam	-	-	2 (<1 %)
Haloperidol	51	10 %	2 (<1 %)
Thiopental	-	-	2 (<1 %)
Type of administration			
i.v. bolus	209	42 %	36 (7 %)
i.v. continuously	253	50 %	36 (7 %)
s.c. bolus	100	20 %	36 (7 %)
s.c. continuously	56	11 %	36 (7 %)

Midazolam was most frequently used for sedation (*n* = 395, 79 %) followed by lorazepam (*n* = 66, 13 %), haloperidol (*n* = 51, 10 %), levomepromazine (*n* = 17, 3 %), diazepam (*n* = 14, 3 %) and propofol (n = 13, 3 %) (Table 2). The majority of patients (*n* = 443, 88 %) received only one drug; 53 patients (11 %) received two drugs. Women received more than one drug for sedation (*p* < 0.004) significantly more often than men. No statistical difference was noted in regard of other clinical variables such as age, diagnosis, and the overall duration of hospitalization.

The routes of administration of sedation were as follows: continuous intravenous (i.v.) administration in 253 patients (50 %), i.v. bolus in 209 (42 %), continuous subcutaneous (s.c.) administration in 56 (11 %), and an s.c. bolus in 100 patients (20 %) (Table 2).

Three hundred nineteen patients (68 %) received medication for sedation via one route of administration, whereas 146 (31 %) received the medication via two routes.

A significant increase in dose from the first to the last 24 h was only registered for midazolam (median drug dose in mg: 15 vs. 24, *p* ≤ 0.001); no significant dose increase was noted for any other drug.

## Discussion

This present study reveals the first insight about the current prevalence and practice of sedation in Austrian palliative care wards and mobile care teams. The following findings of our study deserve particular attention.

To our knowledge to date this is by far the largest study conducted on sedation at the end of life [1, 24]. Twenty-three of 31 (74 %) Austrian palliative care wards and mobile care teams contributed to this study. As these wards and mobile care teams are dedicated palliative care units involved in a nation-wide project on the development of palliative care in Austria, with identical structural requirements and procedural standards, our data provide a clear picture of palliative sedation in Austria. All mobile palliative care units who participated in this study were associated with a hospital based palliative care ward providing access to a multidisciplinary care team. According to the Austrian palliative care system patients have the possibility to receive mobile care according to their personal preferences as well as prevailing mobile care disposability.

The rate of sedation registered in our cohort (21 %) is at the lower end of sedation rates reported previously in a systematic review by Maltoni et al. [24]. However, sedation rates varied very markedly in the participating study centers (range 0–50 %). As in Austria for the use of palliative sedation no stringent guidelines exist, this variation is indicative of a lack of national consensus in this sector. Nation-wide recommendations will be needed in the near future in order to enhance safety in clinical practice.

As reported in the published literature, pain, dyspnea, agitated delirium, and anxiety are among the most common symptoms of cancer patients approaching the end of live [24, 25]. Overall, the prevalence of refractory symptoms requiring sedation ranges from 10 to 50 %, with a median estimate of 20–30 % [24, 26, 27] comparable to our data from Austrian palliative care units (21 %). Consistent with previous findings, delirium was the most common indication for palliative sedation followed by existential distress, dyspnea and pain [24]. Furthermore, as reported previously the majority of all patients are assessed as suffering from more than one symptom [26] and in our study cohort as well close to half to all patients (44 %) were documented with two or three indications for sedation.

Approximately one third of our patients received sedation because of existential distress. As in other reports [17, 21, 28], these patients were significantly younger (*p* ≤ 0.001) than those who were sedated for other reasons, but did not differ in terms of gender and the duration of hospitalization/treatment by a mobile palliative care team.

According to the EAPC, sedation may occasionally be considered in patients with severe psychological symptoms such as refractory depression, anxiety, demoralization or existential distress [6]. However, sedation for the management of existential distress is still controversially discussed in the literature [1, 6, 7]. The term *existential distress* is complex since it includes several symptoms such as feelings of meaninglessness/worthlessness/dependency and isolation, fear or panic of impending death, lack of social support, as well as spiritual issues. Furthermore existential distress may be a dynamic phenomenon in the course of a disease. As it does not necessarily indicate an advanced stage of psychological deterioration, the truly refractory nature of the symptoms cannot be established conclusively [1, 6, 7]. Due to these issues palliative sedation for existential distress, should be considered cautiously, and only after other interventions have been exhausted as determined by a multidisciplinary team [1, 6, 7].

The rather high rate of existential distress in our study may be explained by several factors. First, given the ambiguous definition of existential distress and its clinical symptoms [1, 6, 7], no standardized definition of the term was provided to the centers participating in the study. Secondly, the study was conducted by retrospective chart review, allowing multiple indications for sedation in a single patient. From the clinical perspective it is feasible that existential stress occurs when a specific physical symptom is untreatable. As visible from our data nearly half of all patients (43 %) in our study cohort were documented with two or three indications for sedation; this would account for the high prevalence of existential stress as an indication for sedation in the present study.

Based on the large majority of reports, benzodiazepines remain the most favored class of sedatives in palliative care [1, 6, 24, 28–30]. Midazolam was the drug of first choice in our study population. Effective alternatives such as haloperidol, levomepromazine and propofol [1, 6, 24] played a minor role and were largely used in conjunction with specific indications.

Midazolam, a short-acting benzodiazepine, has anticonvulsant, muscle-relaxing and anxiolytic properties [29]. Psychotropic drugs such as haloperidol or levomepromazine may be more appropriate for the management of delirium, and may be used in combination with benzodiazepines [24, 31]. Propofol is liable to cause rapid unconsciousness [32] and may be an effective last-line option when all other medications have failed [24, 32].

In our patients, women received a single drug for palliative sedation significantly more often (*p* < 0.004*), but no statistical difference was registered for other variables such as age, diagnosis, and the overall duration of hospitalization.

The median duration of sedation in our cohort was within the reported range (two days) [17, 24, 28]; only five patients were sedated for more than three weeks.

We also assessed co-medications during the last three days of the patients’ lives, which provided even greater insight into end-of-life practice in Austrian palliative care units. Overall, the majority of patients who died at a palliative care unit received non-opioids and opioids (53 and 92 %). A substantial number received antibiotics (17 %), prophylaxis for deep vein thrombosis (24 %), artificial hydration (42 %) and nutrition (26 %). Sedated patients received significantly more opioids and artificial hydration, but less artificial nutrition.

Importantly, opinions, practices and decisions regarding the administration of hydration and/or artificial nutrition vary widely in the published literature. These should be viewed independent of the decision for or against palliative sedation [1, 6, 7]. The variance of opinions and practices probably reflects the heterogeneity of patients, caregivers, and clinicians. Consensus must always be achieved together with the patient and/or family members, based on the patient’s best interests [1, 6].

In conformity with other studies [24, 28], the median number of days between admission to a palliative care ward (mobile palliative care team) and death in our cohort did not differ significantly between sedated vs. non-sedated patients (10 vs. 9 days; *p* = 0.491). Importantly, in a further subgroup analysis to exclude the potential bias of sedation level (continuous vs. intermittent), no difference was observed between patients receiving continuous and intermittent sedation regarding the number of days of hospitalization until death (*p* = 0.259). Sedation may therefore be considered an appropriate clinical intervention to relieve terminally ill patients of their intractable symptoms. To the best of our knowledge, it does not exert a negative impact on the patient’s survival.

### Limitations and strengths of the study

Undoubtedly, the retrospective design of our study leaves many important questions unanswered in regard of sedation at the end of a patient’s life.

Owing to the retrospective design, this study provides no more than a rough estimate of the effect of palliative sedation on actual survival and thus cannot reliably prove or reject the life-shortening effect of sedation. A randomized controlled trial would be needed to address this question [24, 28]. However, a trial design of this nature would leave the control group without a treatment option in the event of intractable symptoms, and would therefore be ethically unjustifiable [1, 24].

Another important limitation of our study is the fact that no operational definition on the term *existential distress* was provided to the participating study centers. Besides, the option of main and secondary indications was not taken into account because the units were permitted to use several indications for sedation at a time.

As mentioned earlier, we defined sedation therapy at the end of life as any type of sedation initiated in the last two weeks of a patient’s life and given continuously until death (minimal duration one hour), or as intermittent sedation in the last two weeks of a patient’s life for a duration of more than 24 h, even when the sedation was not administered when the patient died. Further guided instructions concerning deep continuous and partial intermittent sedation were not provided by the study team; these aspects were left to the discretion of the treating physician.

Long-term observation of the timing of co-medications was beyond the scope and feasibility of the retrospective study design. For reasons of practicability we only registered the presence of co-medication in the last three days before death. Besides, important issues relating to palliative sedation, such as decision-making, the intensity of sedation, and the incorporation of family and team members in the decision-making process could not be addressed.

Our study monitored the use of sedatives at the end of patients’ lives in palliative care units in Austria, but deliberately did not use the term ‘palliative sedation’ in the study protocol because many palliative care units employ sedation at the end of life with the intent of palliative sedation, but do not have explicit comprehensive protocols for palliative sedation. Since the study protocol was designed to exclude other indications for sedation at the end of life (such as insomnia), but asked for specific sedation practices typically used for palliative sedation, we conclude that the data do reflect actual palliative sedation practice at these units.

The strengths of the study include the large number of studied patients, the high rate of recruitment at Austrian palliative care wards and mobile care teams, and the small number of incomplete data. Our database was derived from palliative units, which have identical structural requirements and procedural standards, and therefore yielded a homogenous study sample.

## Conclusion

To date this is the largest study on sedation at the end of life and provides preliminary insights into the prevalence of, and indications for, palliative sedation in Austria. It supports previous data which suggest that palliative sedation, when properly administered, is an appropriate therapeutic procedure that does not shorten the period of time from admission to a palliative care ward/mobile care team until death. In order to minimize variation and enhance safety in clinical practice, implementation of a nation-wide guideline for the use of palliative sedation will be developed in Austria.

### Availability of data and materials

All data and material related to the manuscript have been archived by the first and last author (Sophie Schur and Herbert Watzke) at the Medical University of Vienna**.**

## Abbreviations

CRF: case report form
DVTP: deep venous thrombosis prophylaxis
i.v.: intravenous
IQR: interquartile range
PS: palliative sedation
s.c.: subcutaneous
